# Kinetics, Thermodynamics and Mechanism of Enzymatic Degradation of Zearalenone in Degummed Corn Oil

**DOI:** 10.3390/toxins15010019

**Published:** 2022-12-28

**Authors:** Chenwei Zhao, Pengkai Xie, Jun Jin, Qingzhe Jin, Xingguo Wang

**Affiliations:** School of Food Science and Technology, Jiangnan University, Wuxi 214122, China

**Keywords:** zearalenone, hydrolase, degummed corn oil, kinetics, thermodynamic, mechanism

## Abstract

The kinetics and thermodynamics of the enzymatic degradation of zearalenone (ZEN) in degummed corn oil were investigated by analyzing the impacts of temperature, pH, ZEN hydrolase dosage and ZEN concentration on the initial reaction rate. The kinetic study found that the maximum reaction rate was 0.97 μmol × kg^−1^ min^−1^, the Michaelis constant (Km) was 11,476 μmol × kg^−1^ and the Michaelis equation was V = 0.97[S]/(11,476 + [S]). The thermodynamic study showed that the activation energy (Ea) was 70.37 kJ·mol^−1^, the activation enthalpy change of the reaction (ΔH) > 0, the free energy of activation (ΔG) > 0 and the activation entropy change (ΔS) < 0, indicating the reaction could not be spontaneous. The reaction mechanism of ZEN was studied by a hybrid quadrupole orbitrap mass spectrometer. It was found that ZEN first generated the intermediate G/L/D/W-ZEN+H_2_O, followed by generating the intermediate W-ZEN-H_2_O under the action of a degrading enzyme. Then, the lactone bond was opened to produce C_18_H_24_O_6_, and finally the decarboxylation product C_17_H_24_O_4_ formed automatically.

## 1. Introduction

Zearalenone (ZEN), a non-steroidal estrogen mycotoxin, is mainly produced by *Fusarium roseum*, *Fusarium graminearum*, *Fusarium tricinum* and other *Fusarium* microorganisms during their secondary metabolism and is the most widely contaminated mycotoxin in the world [1,2]. Corn is easily polluted by ZEN in the process of planting and harvesting [3]. As ZEN is a benzoic acid lactone compound and exhibits a certain lipophilicity, it is very easily transferred into crude corn oil during corn oil production. The highest content of ZEN in corn crude oil was more than 8000 μg × kg^−1^ [4]. Zearalenone has a similar phenolic dihydroxyacetone structure to β-Estrogens, so it can competitively bind to estrogen receptors in the body after entering humans and animals, thus causing reproductive toxicity, genotoxicity, carcinogenicity and immunotoxicity [5,6,7].

Rapid and effective detoxification methods have attracted increasing attention due to the huge economic losses and health risks caused by ZEN pollution. At present, the detoxification methods of ZEN can be divided into three categories: physical, chemical and biological methods. However, the physical and chemical methods have defects such as incomplete detoxification, the loss of some small molecular nutrients, secondary pollutants and low detoxification efficiency [8,9,10,11,12]. Biological enzymatic detoxification has become a research hotspot because of its irreversible reaction process, mild reaction conditions, no secondary pollutants, low price and many other advantages [12]. Zearalenone hydrolase (ZHD) has the advantages of a clear detoxification mechanism, high degradation activity and no medium required for reaction, showing good application prospects [13].

In 2002, Takahashi Ando et al. successfully expressed the lactone hydrolase encoding gene ZHD101 from *Clonostachys rosea* IFO7063 in *Escherichia coli* [14]. Since then, ZHD101 has gradually become a research hotspot and has been successfully expressed in a variety of hosts including *Escherichia coli* [14], *Pichia pastoris* [15], *Saccharomyces cerevisiae* [16] and *Lactobacillus reuteri* [17]. To obtain ZEN lactone hydrolase with better enzymology properties, some other microbial lactone hydrolases, such as ZHD607 [15] from *Phylophora americana* and *Neurospora crassa* ZENC [18], zhd518 [19] from *Rhinocladiella machinenziei*, CbZHD [20] from *Cladophialophora bantiana* and ZHD607 [15] from *Phylophora americana*, have been gradually excavated and studied.

**Figure 1:**
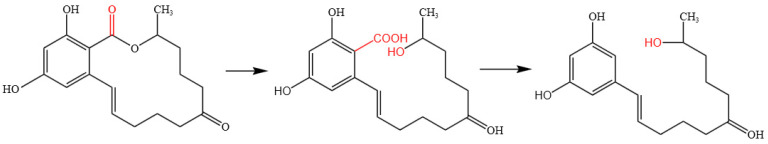
The mechanism of ZHD.

In 2002, Takahashi Ando et al. carried out a thorough study on the mechanism of ZEN degradation catalyzed by lactone hydrolase ZHD101 (as shown in Figure 1) and found that lactone hydrolase can catalyze the cleavage of lactone bonds in ZEN molecules, opening the lactone ring, producing a molecule of carboxyl and a molecule of hydroxyl, and then spontaneously decarboxylating to form a completely non-toxic degradation product (1-(3,5-dihydroxybenzene-10′-hydroxy-1′-trans undecene-6′-one)). A molecule of carbon dioxide is released at the same time [14]. The catalytic mechanism of the enzyme is mainly the result of the joint action of amino acid residues. During the catalytic process, intermediate products bound with amino acids will be formed [21].

It has been reported that ZEN removal from corn oil by enzyme is an environmentally friendly and efficient method [22]. However, the kinetics and mechanism of reaction in crude corn oil have not been clarified. In this study, the kinetics, thermodynamics and mechanism of the enzymatic degradation of zearalenone in degummed corn oil (DCO) were investigated. The structure of the degradation products was identified to explore its reaction mechanism, so as to provide a theoretical basis for the removal of ZEN from corn oil by ZHD.

## 2. Results and Discussion

### 2.1. Effects of pH on Initial Reaction Rate

**Figure 2:**
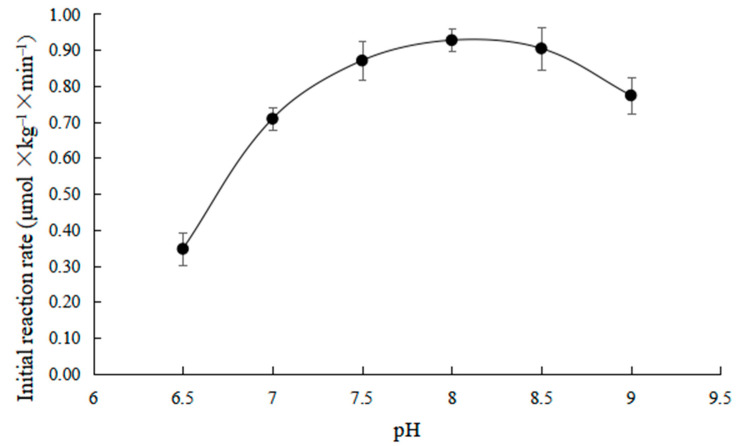
Effects of pH on initial rates of ZEN degradation.

As shown in Figure 2, the effects of different pH on the initial reaction rate were investigated at 35 °C and the ZHD dosage was 30 mg × kg^−1^.

It can be seen from Figure 2 that the initial reaction rate changes in a bell shape with the increase in pH. When the pH was in the range of 7.5–8.5, the initial reaction rate was the highest, which is related to the dissociation state of the active group of the enzyme in different acid-base systems. The combination of the active group of the enzyme and ZEN is in the best state when the pH is 7.5–8.5, making the reaction rate faster. When the pH is higher or lower than the value, the action of the acid or alkali causes the dissociation of the active group of the enzyme to change into an inactive state, thus reducing the reaction rate [23].

### 2.2. Effects of ZHD Dosage on Initial Reaction Rate

**Figure 3:**
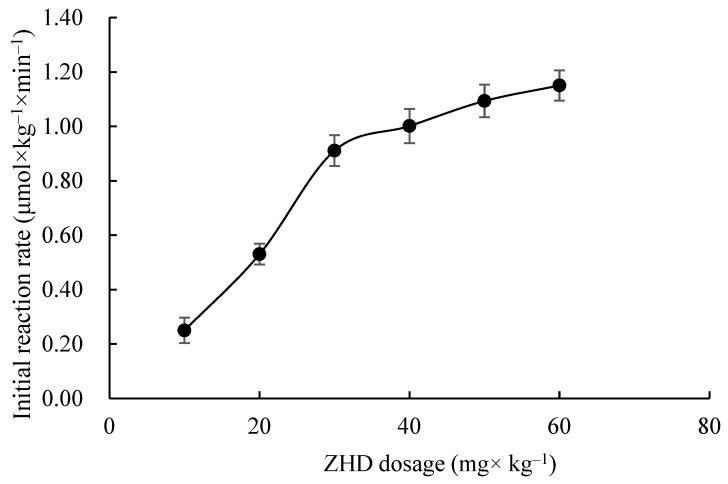
Effects of ZHD dosage on initial rate of ZEN degradation.

As shown in Figure 3, the effect of different ZHD dosages on the initial reaction rate was investigated at 35 °C and pH 8.0.

It can be seen from Figure 3 that the initial reaction rate increases linearly with the increase in the ZHD dosage and then tends to slow down. During enzyme catalysis, the enzyme firstly combines with ZEN to form an intermediate complex. When the enzyme concentration is low, the reaction rate is slow. With the increase in the enzyme concentration, the rate increases in proportion. When the enzyme concentration is large, the enzyme changes from fully saturated to excessive. Under the condition of constant substrate concentration, the reaction rate does not change much [24].

### 2.3. Effects of ZEN Concentration on Initial Reaction Rate

**Figure 4:**
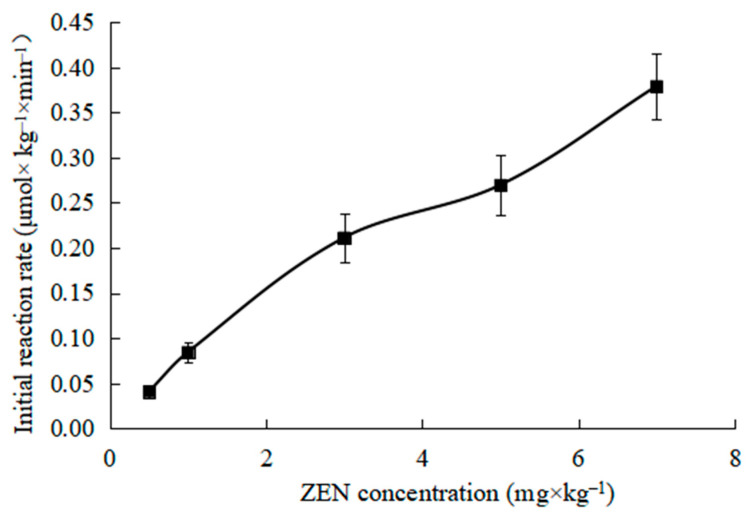
Effect of ZEN concentration on initial rate of ZEN degradation.

As shown in Figure 4, the effect of different ZEN concentrations on the initial reaction rate was investigated at 35 °C and pH 8.0 and the ZHD dosage was 30 mg × kg^−1^.

Zearalenone degradation by ZHD is a hydrolysis reaction, and the reaction substrate is ZEN and water [25]. However, since the concentration of water changes little during the reaction process, it can be treated as a constant, so the reaction can be regarded as a single substrate enzyme catalyzed reaction. It can be seen from Figure 4 that when the enzyme concentration is constant, the initial reaction rate catalyzed by the enzyme increases in a positive proportion with the increase in the substrate concentration, showing a first-order reaction.

### 2.4. Enzyme Reaction Kinetics

**Figure 5:**
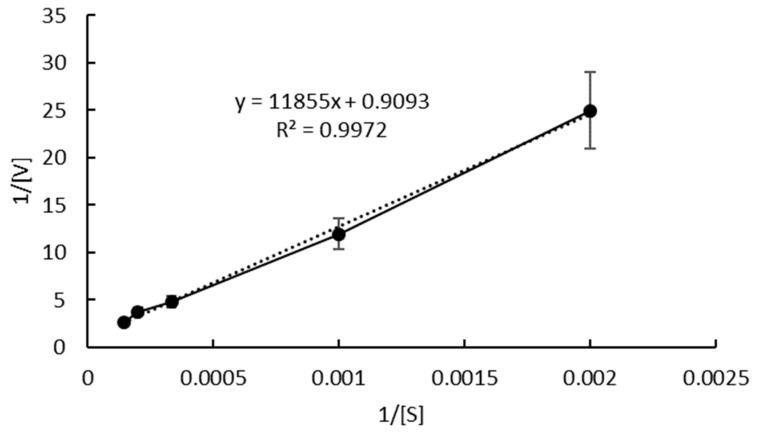
Reciprocal initial reaction rate versus reciprocal ZEN concentration.

The Lineweaver Burk method is used for drawing Figure 4, and the results are shown in Figure 5.

According to the Lineweaver Burk method, the intersection point with the Y axis is Vm^−1^, and the intersection point with the X axis is −Km^−1^. Fitting the obtained curve, the equation (y = 11,626x + 0.9355) is obtained, so that Vm is 1.07 μmol × kg^−1^ × min^−1^ and Km is 12,428 μmol × kg^−1^. Thus, the Michaelis equation of this reaction is V = 1.07[S]/(12,428 + [S]).

The Wilkinson statistical method includes two steps of nonlinear multiplication and Taylor expansion as shown in Table 1 and Table 2. Figure 5 is solved by the Wilkinson statistical method. As can be seen from Table 1, Δ = αε − γδ = 3.4667 × 10^−12^, Vm_0_ = (βε − δ^2^)/Δ = 0.9538 μmol × kg^−1^ × min^−1^, Km_0_ = (βγ − αδ)/Δ = 11,052 μmol × kg^−1^. Vm_0_ and Km_0_ are the estimated values of the maximum reaction rate and Michaelis constant, respectively.

The f and f` in Table 2 are calculated according to the following formula:

(1) $$f=\frac{V_{m0}\left[S\right]}{K_{m0}+\left[S\right]}$$

(2) $$\mathrm{f`}=\frac{-V_{m0}\left[S\right]}{{\left(K_{m0}+\left[S\right]\right)}^{2}}$$

According to Table 2, the following results can be obtained that Δ` = α`β`−γ`2 = 3.5299 × 10^−12^, b_1_ = (β`δ`−γ`ε`)/Δ` = 1.01698, b_2_ = (α`ε`−γ`δ`)/Δ` = 434.9822, so that V_max_ = V_m0_ × b_1_ = 0.97 μmol × kg^−1^ × min^−1^, K_m_ = K_m0_ + b_2_/b_1_ = 11,476 μmol × kg^−1^.

The final Michaelis equation calculated by the above statistical method is V = 0.97[S]/(11,476 + [S]).

There is a certain difference between Vm and Km calculated by the Lineweaver buck method and the Wilkinson statistical method. The Wilkinson statistical method is considered to be reliable [26]. Therefore, the Michaelis equation obtained by the Wilkinson statistical method is finally selected as the final result.

There is a certain gap between Km and V_max_ obtained in this study and the data reported by other scholars [18]. Km reflects the affinity between the enzyme and substrate. The larger the Km, the smaller the affinity between the enzyme and the substrate, and the higher the required substrate concentration when the reaction rate reaches 1/2 of the maximum reaction rate [27]. The substrate ZEN in this study is dissolved in the oil phase, while the enzyme is dissolved in the water phase. The two phases’ immiscibility requires a large substrate concentration to increase the contact probability between the enzyme and the substrate. Therefore, the Km obtained in this study is larger than in others.

### 2.5. Effects of Temperature on Initial Reaction Rate

**Figure 6:**
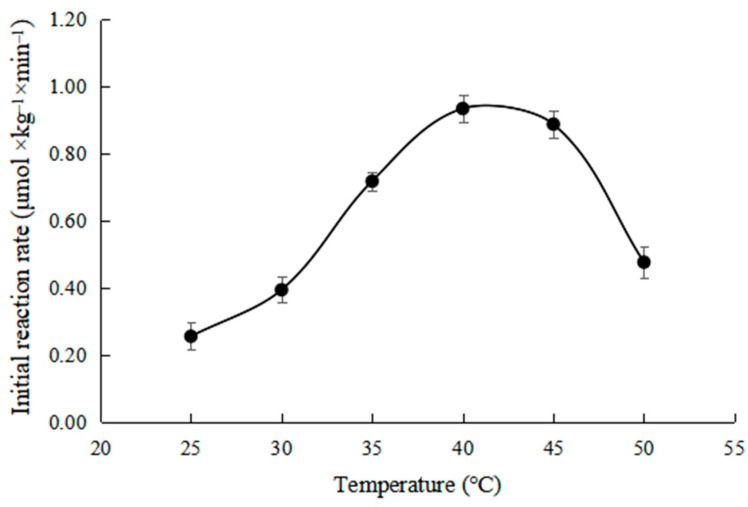
Effect of temperature on initial rate of ZEN degradation.

As shown in Figure 6, the effect of different temperatures on the initial reaction rate was investigated at Ph 7.5, ZHD dosage 30 mg × kg^−1^.

It can be seen from Figure 6 that the initial reaction rate increases first and then decreases with the increase in temperature. This is because the influence of temperature on the enzyme reaction rate includes two aspects. On the one hand, the material thermodynamic movement intensifies with increasing temperatures, and the enzyme reaction rate increases because the contact probability between substrate molecules increases. On the other hand, the enzyme protein denaturates gradually with the increase in temperature, and the enzyme activity decreases, resulting in a decrease in the reaction rate [28]. The temperature when the rate reaches the highest point is the optimal reaction temperature of the enzyme. It can be seen from Figure 2 that the enzyme reached the optimum reaction temperature at about 40 °C. The result was consistent with the conclusion reported by Wang [19].

### 2.6. Determination of Thermodynamic Parameters

**Figure 7:**
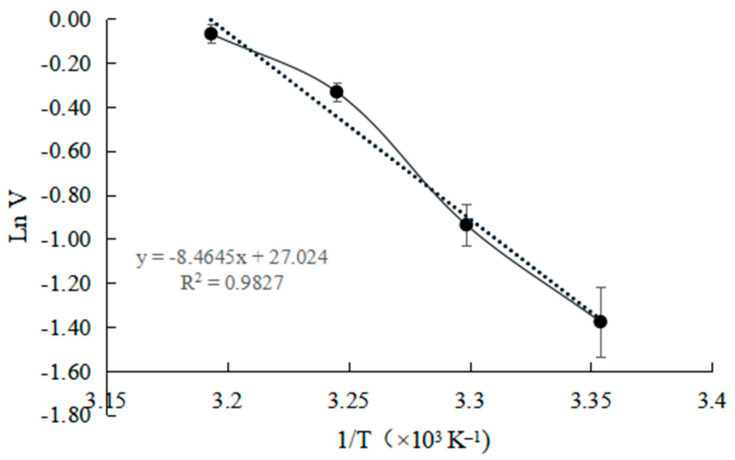
Arrhenius plot to calculate activation energy Ea of ZHD.

It can be seen from Figure 6 that, in the range of 25–40 °C, the initial reaction rate is linear with the increase in temperature. As shown in Figure 7, the Arrhenius plot was applied to calculate the activation energy (Ea). The activation energy (Ea) is 70.37 kJ × mol^−1^.

At present, there is no data report on the Ea of ZHD, but the Ea of lactone hydrolase for other use is 56.8 kJ mol^−1^, which is little lower than that calculated in this study [29]. The slightly higher activation energy (Ea) indicates that the formation of the enzyme substrate complex requires more energy. This feature makes the industrial application of ZHD require more energy supplement, and this feature should be taken into account in the process application.

As shown in Table 3, the thermodynamic parameters for ZEN degradation by ZHD were calculated according to Equations (4)–(6).

It can be seen from Table 3 that ΔH and ΔG are more than 0, and ΔS is less than 0, indicating that the reaction cannot be spontaneous. The enthalpy change of activation usually represents the energy transferred from the outside to make the active site of the enzyme fit with the substrate. If the value is greater than 0, it indicates that the reaction is endothermic [30]. ΔS < 0 indicates that the reaction is a process of entropy reduction [31], which is because ZEN is dissolved in oil and needs to be exchanged to the reaction interface, thus losing some degrees of freedom. ΔG > 0 indicates that the reaction is not spontaneous [32], and it needs to increase the oil-water contact area by providing energy.

### 2.7. Mechanism of ZEN Hydrolase

**Figure 8:**
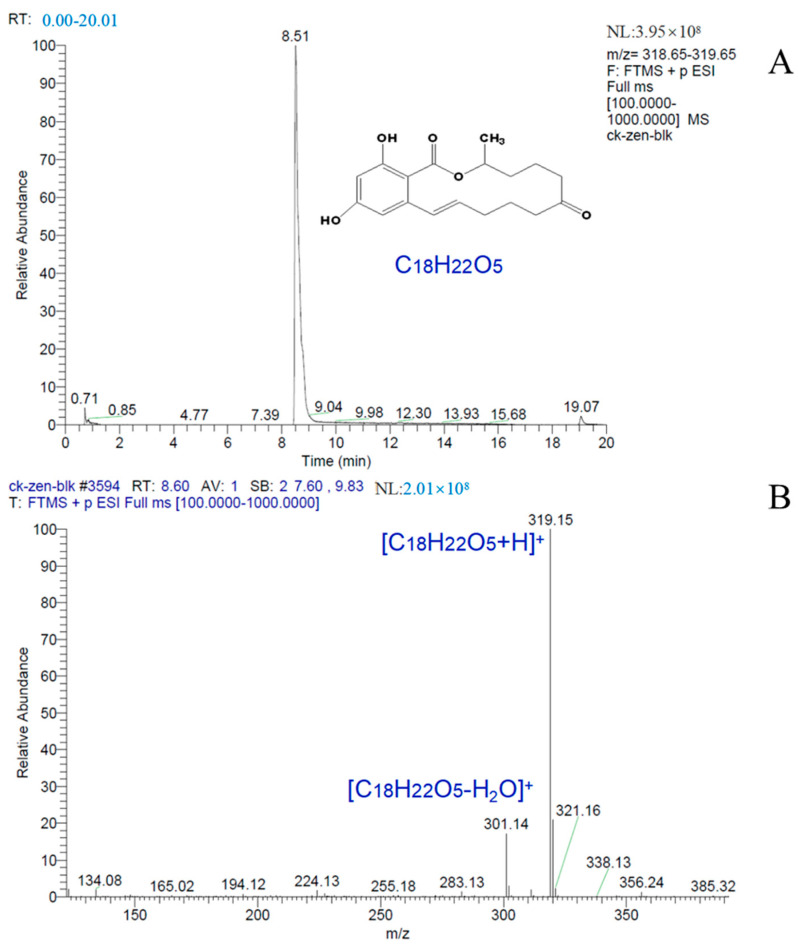
HPLC-MS TIC of ZEN in DCO before reaction. ((**A**), the chromatogram of ZEN, and (**B**), the mass spectrum of ZEN).

As shown in Figure 8, the ZEN in the DCO before the reaction was analyzed by LC-MS.

It can be seen from Figure 8 that ZEN has a peak retention time of 8.51 min in ESI+ ion mode, and can form the [C_18_H_22_O_5_+H]^+^ parent ion (*m*/*z* 319.15) and [C_18_H_22_O_5_-H_2_O]^+^ fragment ion (*m*/*z* 301.14) under mass spectrometry conditions. Francesca et al. [33] reported that the above two ions are characteristic ions of zearalenone LC-MS, and the results are consistent with the literature.

**Figure 9:**
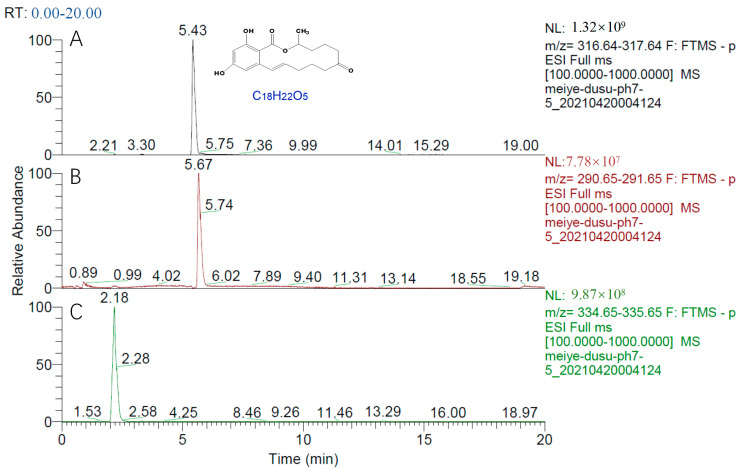
HPLC-MS chromatogram of zearalenone (**A**) and zearalenone hydrolysate (**B**,**C**) (ES-scan mode).

The chromatogram of the negative ion scanning results of the enzymolysis reaction products is shown in Figure 9, wherein Figure 9A shows the chromatographic peak of ZEN (5.43 min); Figure 9B,C show that ZEN in DCO generates product B (2.18 min) and product C (5.67 min), respectively, under the condition of enzymatic hydrolysis.

**Figure 10:**
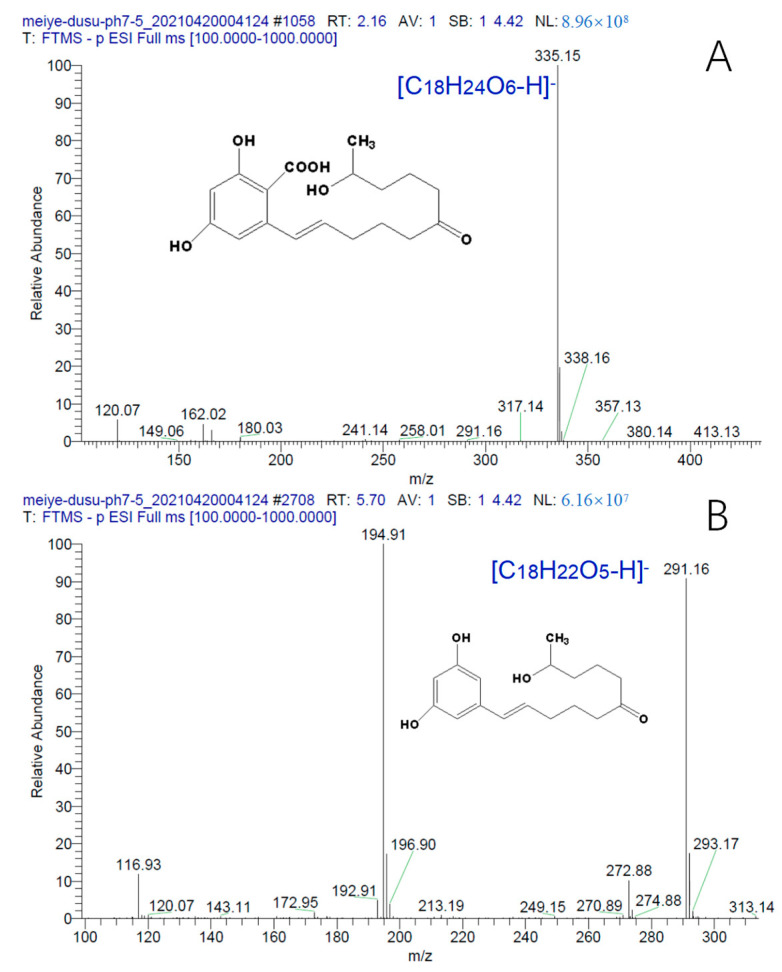
HPLC-MS mass spectrum of zearalenone hydrolysates (ESI-scanning mode). ((**A**), HPLC-MS mass spectrum of water-added product and (**B**), HPLC-MS mass spectrum of decarboxylation product).

The mass spectra of B and C in Figure 9 are shown in Figure 10A and Figure 10B, respectively. The two products formed [C_18_H_24_O_6_-H]^−^ (*m*/*z* 335.15) and [C_18_H_22_O_5_-H]^−^ (*m*/*z* 291.16) mother ions, respectively.

It can be seen from Figure 10 that the molecular weights of the two degradation products of ZEN are 335.15 and 291.16, respectively. The molecular weight of A is 18 higher than that of ZEN, which is the molecular weight of water. This indicates that the hydrolysis of the lactone bond of ZEN opens and becomes carboxyl and hydroxyl groups. The molecular weight of B (43.99) was less than that of A, which is the molecular weight of carbon dioxide, indicating that product A (water-added product) further reacts to remove the carboxyl group generated by hydrolysis to obtain product B (decarboxylation product).

**Figure 11:**
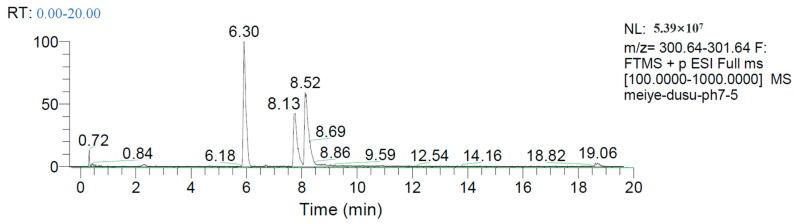
HPLC-MS chromatogram of zearalenone hydrolysate (ESI + scan mode).

**Figure 12:**
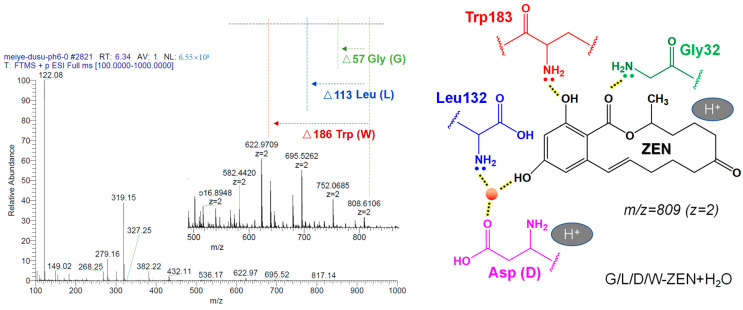
Identification of hydrolysates with retention time of 6.30 min (ESI + scan mode).

**Figure 13:**
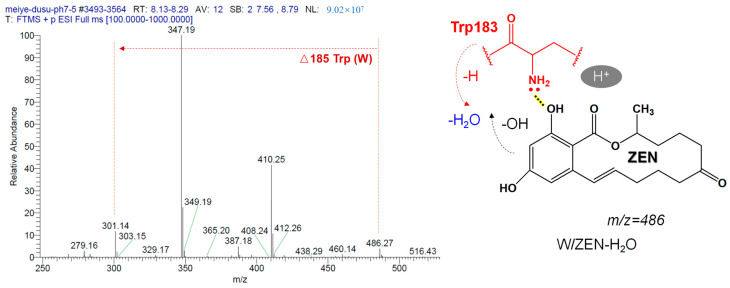
Identification of hydrolysates with retention time of 8.13 min (ESI + scan mode).

The chromatogram of the positive ion scanning results of the enzymolysis reaction products is shown in Figure 11. It can be seen from Figure 8 that the chromatographic peak with a retention time of 8.52 min represents ZEN. The chromatographic identification process with 6.30 min and 8.13 min retention times is shown in Figure 12 and Figure 13.

As shown in Figure 12, the mass spectrum with a retention time of 6.30 min implies that the parent ion of the substance is double-charged *m*/*z* 808.61. Further, the difference is calculated by the fragment ions (*m*/*z* 752.06, *m*/*z* 695.52, *m*/*z* 622.97) to get exactly three amino acid residues, namely glycine (Gly, G), leucine (Leu, L) and tryptophan (Trp, W). The above amino acid residues correspond to neutral losses Δ57, Δ113 and Δ186, respectively. Further calculation of the molecular weight shows that the substance also contains aspartic acid residues (Asp, D). As aspartic acid belongs to an acidic amino acid, it is easy to lose a water molecule (H_2_O) and add an additional positive charge under the condition of collision-induced dissociation (CID), so the whole molecular system shows a double charge (*m*/*z* = 809, z = 2). Finally, based on the research results of Qi et al. [21], it is found that Gly32, Leu132 and Trp183 form strong hydrogen bonds in different parts of ZEN, especially the hydrogen bond force between water molecules and a variety of amino acids near the phenol hydroxyl group. It can be inferred that the substance is G/L/D/W-ZEN+H_2_O, and it is determined as the intermediate product I of the enzymatic hydrolysis reaction.

As shown in Figure 13, the mass spectrum with a retention time of 8.13 min implies that the parent ion of the substance is *m*/*z* 486.27. Further, by calculating the difference with the ZEN common characteristic fragment ion (*m*/*z* 301.14), we can get exactly one amino acid residue, tryptophan (Trp, W) in turn, with a neutral loss of Δ185. It may be that Trp contributes a proton H to combine with -OH of ZEN to lose molecule water. Finally, we can infer that the substance is W-ZEN-H_2_O, and it is determined as the intermediate product II of the enzymatic hydrolysis reaction.

It is reported that the structure of ZEN hydrolase can be divided into two parts: the folding center domain and the cap structure of the hydrolase [34]. A large groove is formed at the junction of the two structures, which is proved to be the substrate binding site by the substrate complex structure. The benzene ring part of the substrate ZEN is mainly fixed by hydrogen bonds, and the lactone ring part is mainly bound to the active center by hydrophobic force.

**Figure 14:**
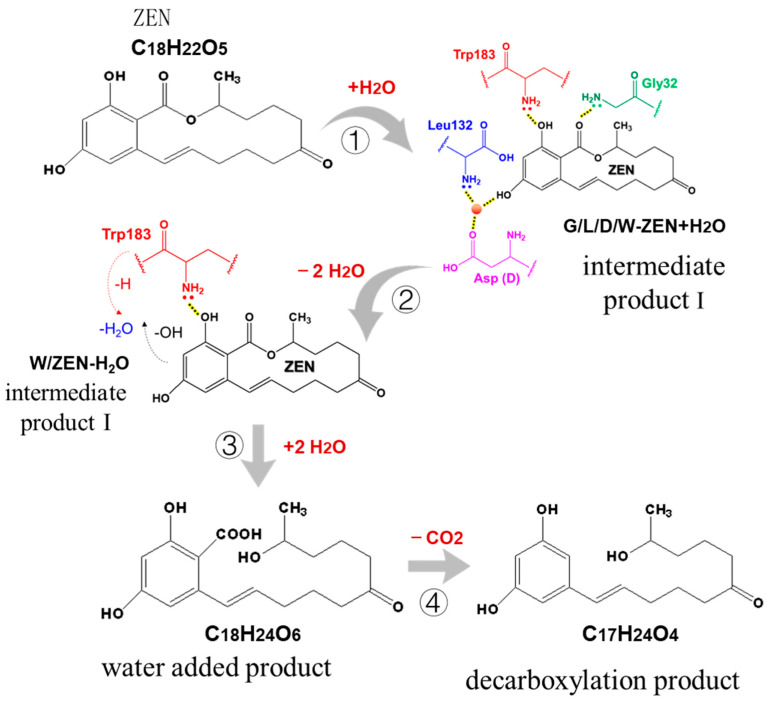
Degradation pathway of ZEN by ZHD.

The pathway of ZEN degradation by ZEN hydrolase is shown in Figure 14.

It can be inferred from Figure 14 that, under the action of ZEN hydrolase, a water molecule binds to the 4-hydroxy group of the benzene ring of ZEN to form the intermediate product I, so that ZEN and the enzyme form a hydrogen bond fixation. After that, glycine, leucine and aspartic acid are removed, tryptophan is retained, and intramolecular dehydration forms intermediate product II. Then, two water molecule bond intermediate product II tryptophan is removed, and the lactone bond is opened to form water-added product C_18_H_24_O_6_. Finally, the water-added product loses a carboxyl group automatically to form the decarboxylation product C_17_H_24_O_4_.

## 3. Conclusions

The kinetics and mechanism of the enzymatic degradation of ZEN in DCO were investigated, and the following conclusions were obtained.

(1) The initial reaction rate increases firstly and then decreases with the increases in temperature and pH. It rises in a straight line and then tends to slow down with the increase in the dosage of enzyme added. Moreover, with the increase in substrate concentration, the initial reaction rate presents a positive proportion, which is a first-order reaction.

(2) The kinetic study found that the maximum reaction rate was 0.97 μmol × kg^−1^ × min^−1^, the Michaelis constant (Km) was 11,476 μmol × kg^−1^ and the Michaelis equation was V = 0.97[S]/(11,476 + [S]).

(3) Thermodynamic study shows that the activation energy (Ea) is 70.37 kJ·mol, the activation enthalpy change (ΔH) > 0, the free energy (ΔG) > 0, the activation entropy change (ΔS) < 0 and the reaction cannot be spontaneous.

(4) The reaction mechanism of ZEN was studied by liquid chromatography mass spectrometry. It was found that ZEN first produced the intermediate G/L/D/W-ZEN+H_2_O, and then produced the intermediate W-ZEN-H_2_O under the action of the degrading enzyme. Then, the lactone bond was opened to produce the water-added product C_18_H_24_O_6_, and finally the decarboxylation product C_17_H_24_O_4_ formed automatically. 

## 4. Materials and Methods

### 4.1. Materials

Degummed corn oil (DCO) was obtained from Hebei Yufeng bio-engineering Co., Ltd. in Xingtai, China, and its contents of ZEN were 554 μg × kg^−1^. Zearalenone hydrolase was obtained from Rongvezyme Preparation Co., Ltd., Zhuhai, China; the enzyme activity was 243 U × g^−1^, which was measured according to the methods of Wang [19]. Zearalenone standard was purchased from Sigma–Aldrich (St. Louis, MO, USA). Tris, hydrochloric acid and other solvents and chemicals were all of analytical grade (Sinopharm Holdings Ltd., Shanghai, China).

### 4.2. Methods

#### 4.2.1. Detection of ZEN Content

The concentrations of ZEN in the corn oil before and after processing were determined by high-performance liquid chromatography with a fluorescence detector (Waters 2695, Waters Co., Ltd., Milford, MA, USA) in accordance with GB 5009.209-2016 (National Standard of the People’s Republic China, 2016).

Separation was conducted by a C_18_ column (4 µm particle size, 150 mm × 4.6 mm, Waters Co., Ltd., Milford, MA, USA) at 25 °C, and the injection volume was set as 100 µL. A mobile phase consisting of acetonitrile-water-methanol [46:46:8, *v*/*v*/*v*] was used at a flow rate of 1 mL × min^−1^.

#### 4.2.2. Determination of the Initial Reaction Rate of ZEN Hydrolase 

Degummed corn oil (500 g), 0.1 mol/L Tris HCI buffer (3 mL, pH 7.5) and 15 mg ZEN hydrolase were added to a beaker. Then, the sample was added to a high-speed shear machine (IKA ULTRA-TURRAX UTL2000, IKA Co., Ltd., Staufen, Germany) to mix the samples at 5000 r/min for 2 min. After that, the mixed sample (100 g) was put into the enzyme reactor at a certain temperature. After 10 min, the temperature was raised to 95 °C to terminate the reaction. Then, the sample was separated by a centrifuge at 10,000 r/min for 10 min. The upper oil layer was dried at 0.09 mPa and 80 °C. Finally, the content of the ZEN of the sample was determined by HPLC.

The initial reaction rate was calculated by the equation

(3) $$V=\frac{\left(M_{0}-M_{1}\right)}{t\times M}\times 10^{-6}$$

where V is the initial reaction rate (μmol × kg^−1^ × min^−1^); M_0_ is the content of ZEN in DCO after the reaction (μg × kg^−1^); M_1_ is the content of ZEN in DCO before the reaction (μg × kg^−1^); t is the reaction time (10 min); M is the molar mass of ZEN (318 g × mol^−1^).

#### 4.2.3. Determination of Kinetic Constants and Thermodynamic Parameters

To study the effects of pH on the initial reaction rate of ZEN degradation, different buffers were used for pH 6.5–9.0.

To study the effect of ZHD dosage on the initial reaction rate of ZEN degradation, different ZHD dosages were used for 10–60 mg × kg^−1^.

To study the effect of the concentration of ZEN on the initial reaction rate of ZEN degradation, different concentrations of ZEN were used for 500–7000 μg × kg^−1^. Lineweaver-Burk plot and Wilkinson statistical method were applied to calculate the Michaelis constant (Km) and maximum velocity (Vmax).

The effect of reaction temperature on the initial reaction rate of ZEN degradation was investigated at a range of temperatures between 25 and 50 °C. The activation energy (Ea) was calculated from the slope of the Arrhenius plot according to the equation:

lnk = −Ea/(RT)(4)

where k, rate of activation at T; R, gas constant (8.314 J × mol^−1^ × K^−1^); T, the absolute temperature in Kelvin.

The thermodynamic parameters for substrate hydrolysis were calculated according to equation [35].

kcat = (kbT/h) × e(−ΔH/RT) × e(−ΔS/R)(5)

where h, Planck’s constant (6.626 × 10^−34^ J × s); K_b_, Boltzmann’s constant (1.3807 × 10^−23^ J × K^−1^); T, Absolute temperature (K); N, Avogadro’s number (6.02 × 10^23^ mol^−1^); R, Gas constant (8.314 J × mol^−1^ × K^−1^)

ΔH, (enthalpy of activation) =Ea − RT(6)

ΔG, (free energy of activation) = −RT ln (K_cat_ h/K_b_ × T) (7)

ΔS, (entropy of activation) = (ΔH − ΔG)/T(8)

#### 4.2.4. Determination of ZEN Degradation Products by Hybrid Quadrupole Orbitrap Mass Spectrometer

Liquid chromatographic conditions: chromatographic column: Thermo Accucore RP-MS 100 mm × 2.1 mm, particle size 1.8 μm. Column temperature: 30 °C, injection volume: 5 μL. Flow rate: 0.3 mL/min. Mobile phase: positive ion mode: Water phase (A): 0.1% formic acid aqueous solution; organic phase (B): 0.1% formic acid acetonitrile. Negative ion mode: aqueous phase (A): 0.03% ammonia aqueous solution, organic phase (B): 0.03% ammonia acetonitrile. Gradient elution: positive and negative are the same. The gradient ratio of mobile phase is 0 min, 95% A, 5% B, 15 min, 5% A, 95% B, 17 min, 5% A, 95% B, 17.1 min, 95% A, 5% B, 20 min, 95% A, 5% B.

Mass spectrum conditions: Positive ion scanning parameters: Scan type: Full MS, Scan range: 70.0 to 1050.0 *m*/*z*, Resolution: 70,000. Polarity: Positive, AGC target: 1 × 10^6^, Max inject time: 50. Positive ion mode ion source parameters: Sheath gas flow rate: 40, Aux gas flow rate: 5, Sweep gas flow rate: 1, Spray voltage (KV): 3.2, Capillary temperature (°C): 320, S-lens RF level: 55.0. Aux gas heater temperature (°C): 400. Anion scanning parameters: Scan type: Full MS, Scan range: 70.0 to 1050.0 *m*/*z*, Resolution: 70,000, Polarity: Negative, AGC target: 1 × 10^6^, Max inject time: 50. Anion mode ion source parameters: Sheath gas flow rate: 40, Aux gas flow rate: 5, Sweep gas flow rate: 1, Spray voltage (KV): 2.8, Capillary temp. (°C): 320, S-lens RF level: 55.0, Aux gas heater temp. (°C): 400.

### 4.3. Statistical Analyses

All experiments were carried out in triplicate independently and SPSS (version 19.0, SPSS, Inc., Chicago, IL, USA) was used for data analysis. Results are presented as mean ± standard deviation of triplicate measurements.

## Figures and Tables

**Table 1:** Estimation solution by Wilkinson statistical method.

[S]	V	X = V^2^	Y = V^2^/[S]	VX	X^2^	VY	XY	Y^2^
500	0.04	0.001663	3.33 × 10^−6^	6.78 × 10^−5^	2.76 × 10^−6^	1.356 × 10^−7^	5.529 × 10^−9^	1.106 × 10^−11^
1000	0.08	0.007156	7.16 × 10^−6^	0.61 × 10^−4^	5.12 × 10^−5^	6.053 × 10^−7^	5.120 × 10^−8^	5.120 × 10^−11^
3000	0.21	0.044701	1.49 × 10^−5^	0.0095	0.00199	3.150 × 10^−6^	6.660 × 10^−7^	2.220 × 10^−10^
5000	0.27	0.072798	1.46 × 10^−5^	0.0196	0.0053	3.928 × 10^−6^	1.059 × 10^−6^	2.119 × 10^−10^
7000	0.38	0.143907	2.06 × 10^−5^	0.0546	0.0207	7.798 × 10^−6^	2.958 × 10^−6^	4.226 × 10^−10^
Σ				0.0844	0.0281	1.561 × 10^−5^	4.741 × 10^−6^	9.189 × 10^−10^
code				α	β	γ	δ	ε

**Table 2:** Exact solution by Wilkinson statistical method.

[S]	[S] + K_m0_	Vm[S]	f	f`	f^2^	f`f	vf	vf`
500	11,551.78	476.9	0.0413	−3.574 × 10^−6^	0.0017	1.277 × 10^−11^	−1.475 × 10^−7^	0.00168
1000	12,051.78	953.8	0.0791	−6.567 × 10^−6^	0.0063	4.312 × 10^−11^	−5.197 × 10^−7^	0.00669
3000	14,051.78	2861.4	0.2036	−1.449 × 10^−5^	0.0415	2.100 × 10^−10^	−2.951 × 10^−6^	0.04305
5000	16,051.78	4769	0.2971	−1.851 × 10^−5^	0.0883	3.420 × 10^−10^	−5.499 × 10^−6^	0.08016
7000	18,051.78	6676.6	0.3699	−2.049 × 10^−5^	0.1368	4.198 × 10^−10^	−7.578 × 10^−6^	0.14030
Σ					0.2745	1.028 × 10^−9^	−1.669 × 10^−5^	0.27189
code				α`	β`	γ`	δ`	ε`

**Table 3:** Thermodynamic parameters for ZEN degradation by ZHD.

Temp. (°C)	Kcat (sec^−1^)	Ea (kJ × mol^−1^)	ΔH (kJ × mol^−1^)	ΔG (kJ × mol^−1^)	ΔS (kJ × mol^−1^ × K^−1^)
25	0.2679	70.37	67.46	70.58	−0.0104
30	0.2569	70.37	67.42	71.91	−0.0148
35	0.2463	70.37	67.38	73.24	−0.0190
40	0.2362	70.37	67.34	74.58	−0.0231

## Data Availability

The data that support the findings of this study are available from the corresponding author upon reasonable request.
